# Precision health equity for racialized communities

**DOI:** 10.1186/s12939-023-02049-4

**Published:** 2023-12-12

**Authors:** Arafaat A. Valiani, David Anderson, Angela Gonzales, Mandi Gray, Lorian Hardcastle, Tanvir C. Turin

**Affiliations:** 1 The Department of History and Global Health Program, University of Oregon, 1288 University of Oregon, Eugene, OR 97403 USA; 2 Department of Community Health Sciences, Cumming School of Medicine, University of Calgary, 3D10, 3280 Hospital Drive NW, Calgary, AB T2N 4Z6 Canada; 3 The Department of Biochemistry & Molecular Biology, Cumming School of Medicine, University of Calgary, HMRB 231, 3330 Hospital Drive NW, Calgary, AB T2N 4N1 Canada; 4 School of Social Transformation, College of Liberal Arts and Sciences, Arizona State University, PO Box 876403, Tempe, AZ 85287-6403 USA; 5 The Department of Sociology, Otonabee College 221.1, Trent University, 1600 West Bank Drive, Peterborough, ON K9J 7B8 Canada; 6 Faculty of Law, University of Calgary, Murray Fraser Hall 3345, 2500 University Drive NW, Calgary, AB T2N 1N4 Canada; 7 The Department of Family Medicine, Cumming School of Medicine, University of Calgary, HSC G012, 3330 Hospital Drive NW, Calgary, AB T2N 4N1 Canada

**Keywords:** Health equity, Race, Intersectionality, Precision health, Precision medicine, Human genomics, Canada

## Abstract

In the last three decades, a cohort of genomicists have intentionally sought to include more racially diverse people in their research in human genomics and precision medicine. How such efforts to be inclusive in human genomic research and precision medicine are modeled and enacted, specifically if the terms of inclusion are equitable for these communities remains to be explored. In this commentary, we review the historical context in which issues of racial inclusion arose with early genome and genetics projects. We then discuss attempts to include racialized peoples in more recent human genomics research. In conclusion, we raise critical issues to consider in the future of equitable human genomics and precision medicine research involving racialized communities, particularly as it concerns working towards what we call *Precision Health Equity* (PHE)*.* Specifically, we examine issues of genetic data governance and the terms of participation in inclusive human genomics and precision health research. We do so by drawing on insights and protocols developed by researchers investigating Indigenous Data Sovereignty and propose exploring their application and adaptation to precision health research involving racialized communities.

## Background

The introduction of precision medicine may offer significant prospects to improve access as a form of preventative and diagnostic care and therefore equity with respect to health care services for racialized communities in Canada. Barriers for racialized communities to access these health innovations, however, still exist because they are significantly underrepresented in genome-wide association studies (GWAS). This is a crucial issue because GWAS data is an important pillar, among others, on which precision medicine interventions relies. Recent studies have striven to design more inclusive studies in human genomics that would produce more representative genomic data of racialized peoples and therefore include disease-associated genetic variations occurring among a diverse range of racialized communities. This insight is an undeniably important advance in this field. We strive to build on this work by exploring whether the terms of such efforts to be inclusive in human genomics research and precision medicine are—in fact—equitable for racialized communities. This commentary aims to outline critical issues to consider in the future of equitable human genomics research and precision medicine involving racialized communities, particularly as it concerns working towards what we call *Precision Health Equity.*

### Main text

The Human Genome Project (HGP) led to significant advances in DNA sequencing technology and bioinformatics analysis software, which catalyzed an explosion in new methodologies and diagnostics that enabled clinical screening at an unprecedented level (i.e. precision medicine). Despite these advances, precision medicine must also contend with issues of exploitation which are foundational in the history of genetics. These instances can include the secondary research of genetic data by academic researchers for purposes (sometimes not even medical in nature) that were not authorized by participating Indigenous or racialized communities, as with cases that unfolded in the 1980s in western Canada. They can also include unintended consequences of stigmatizing the very group researchers seek to genetically screen for a condition; this occurred, for example, in the initial stages of sickle-cell screening programs that focused on Black Californians in the early 1960s. Genetic screening in this time has also exerted eugenic influence on reproductive choices of some African Americans in which a sickle-cell trait was discovered through screening. In all, such experiences have made many racialized communities weary of participating in genetic research (despite reforms that have subsequently been introduced in screening programs). To their credit, contemporary genomicists strive to avoid repeating these past experiences and include hitherto excluded racialized communities in their GWAS that provide the genomic data on which precision medicine depends (as in the case of The Human Pangenome that we specifically examine). This Comment strives to build on these advances, focusing our discussion specifically on inclusion efforts in GWAS regarding complex diseases. In an effort to foreground the recognition of issues concerning equity in this field, we discuss the strategies to advance health equity for racialized communities by introducing the concept of Precision Health Equity. It comprises: first, that research teams develop meaningful and collaborative partnerships, and execute a data-sharing agreement, with racialized communities participating in human genomics research. Second, community-based participatory research tenets, employed in ‘DNA On Loan’ and similar biobanking protocols advanced by studies conducted in Canada (like the Silent Genomes study) and the Aotearoa Variome study (Aotearoa/New Zealand) and genetic studies undertaken by the Native Biodata Consortium (United States). In their own collaborative and specific ways, these studies adopt and adapt Indigenous Data Sovereignty (IDS) protocols to human genomics research that, in our view, propose models by which to equitably include historically marginalized communities in precision medicine.

## Conclusion

While previous IDS initiatives should not be used as a template, those in existence suggest models of how precision medicine might be explored and adapted to studies involving racialized communities in manners that are both inclusive *and* equitable. These models could be adapted to fit and evolve with a diversity of contexts, values and needs of different racialized communities. Stated in the broadest sense, full partnership in a given genetic study and co-stewardship over the biological samples and genetic data derived from genomics research characterize two central tenets towards which Precision Health Equity for racialized communities might work. In our view, these approaches merit further and deeper research in the context of specific human genomics and precision medicine initiatives that seek to include racialized communities.

## Background

Despite the fact that the introduction of human genomics and precision medicine (we use these terms below, as well as *precision health*, interchangeably to avoid repetition) may offer significant prospects to improve accessibility to health care services for racialized communities in Canada, namely because genetic screening is central to precision medicine and it constitutes a form of much-needed preventative care, research about how to ensure that these groups are equitably included is still at a nascent stage. (We employ the term ‘racialized’ critically in this essay to refer to the peoples classified by modern imperial states and their legacy institutions, being reflexive of its colonial origins). By utilizing key technologies to leverage unprecedented scales of patient data (including, but not limited to, comparing genomes, or exomes, with databases of disease-associated genetic markers), precision medicine is expected to offer the possibility of analyzing complex biological and environmental factors in order to precisely tailor clinical decision making (*i.e.* diagnostic and treatment decision) to individual patients (please see Table 1) [1]. In particular, precision medicine has the potential to empower patients to engage in preventative and diagnostic care, potentially improving outcomes in meaningful ways (provided other barriers to accessibility, which are described below, are also simultaneously addressed).

**Table 1:** Defining common tools used to deliver precision medicine

The key technologies driving Precision Medicine involve applying assays and tests that emerged from the research field in molecular biology. They include high-volume sequencing platforms (such as Illumina “next-generation” sequencing), but are not limited to that. Approaches can test for many levels of the molecular biological milieu, including the DNA (i.e. heritable “information” housed in the nucleus), RNA (i.e. the conversion of DNA information into molecules that permeate the cell and are used to construct functional proteins), proteins (i.e. the primary functional molecules of the cell), lipids and metabolites (i.e. other important molecular components of the cell). We briefly define those testing areas here, but emphasize the outsized importance of DNA-based assessments (which are typically needed in order to inform all other levels of evaluation)
Genomics—The assessment of the complete repertoire of heritable DNA; with few exceptions (e.g. cancer), results will be consistent across all cells in one’s body and over time
Exomics—The assessment specifically of the “expressed” sub-set of DNA (estimated at ~ 2% of the whole genome). While this is not as complete as a genomic test, the majority of known disease-causing variation is found in expressed regions, so this is a common economical tool in precision medicine. As with genomic tests, the results should be consistent across cell types and over one’s life span
Transcriptomics—The assessment of RNA, which provides indirect information about what genes are being expressed and to what level. Unlike genomic and exomic tests, these results will not be consistent across cell types in the body nor over time, and so may need to be performed many times and under acute circumstances to reveal the mechanism underlying one’s disease
Proteomics—The assessment of proteins, which provides direct information about what genes are being expressed and about the repertoire of functional molecules in the cell. As with transcriptomics, it will vary across cell types and over time. Critically, while this is a direct assay of functional molecules, it is much more expensive and difficult to perform at scale
Lipidomics—The specific assessment of lipid molecules, which can reveal issues with lipid synthesis pathways that are associated with many specific diseases. As with proteomics, these tests are more expensive and difficult to perform at scale
Metabolomics—The assessment of metabolites more broadly (which may include lipid molecules), used to assess activity of different metabolic biochemical pathways, and can reveal any functional bottlenecks that can contribute to diseases. As with Proteomic and lipidomic tests, they are much harder and more expensive to perform at scale

Precision medicine has emerged out of the Human Genome Project (HGP), which began in the 1990s, and from thousands of genome-wide association studies (GWAS) that have since proliferated based on the resulting genetic maps of the HGP [2]. These GWAS initiatives have formed the foundation of genomic medicine (by identifying associations with diseases which improve disease prediction and that can point the way to potential therapeutic interventions) that is a key, and disproportionate, part of the delivery of precision medicine. GWAS studies that can play a role in preventative care utilize large-scale genome assessments which rely on GWAS studies that identify genetic susceptibility, risk prediction, and disease gene identification, largely using variants that are commonly found in the population. (This should be differentiated from clinical genetic diagnostic testing which typically focuses on identifying variants that cause an individual’s disease (usually rare variants).

A number of studies underline the absence of genomic data on racialized peoples in GWAS studies. For example, Segun Fatumo and their coauthors note that a mere 1.1% of participants in the GWAS Catalog are of African ancestry [3]. Similarly, Nanibaa’ A. Garrison (2019) and their coauthors note that the Genome Aggregation Database (known as gnomAD) “includes reference exome and genome variant information on more than 141,000 individuals globally…but it lacks information on Indigenous people” [4].

This lack of inclusion in GWAS studies is exacerbated by the unrepresentativeness of the human reference genome, that has historically failed to reflect racialized populations—as diverse and differentiated as they are (which in fact fails both racialized and non-racialized populations too). Subsequent efforts to fill the gaps in the Human Genome Project have been certainly undertaken by investigators to create “a reference that is more complete and more representative” as in the case of the Human Pangenome for example. Efforts like these are important because they strive to include racialized populations whose ancestries originate in the global south and north. We hope that more studies will make similar efforts to create a more representative reference which will help to address a fundamental inequity in the foundational studies and data collection which informs precision medicine. Currently, such efforts are being made but they are not consistently part of the methodologies of all GWAS studies, and this lack of coordination or consistent standards continue to disproportionately exclude racialized peoples. Ultimately, the benefits of precision medicine for racialized communities will be in proportion to the degree of their inclusion [5]. If we do not witness more efforts to include racialized peoples in GWAS studies, it is possible that precision medicine could inadvertently exacerbate disparities in health outcomes. If we do see success in these inclusion efforts however, precision medicine has the opportunity to improve these disparities in health outcomes.

Creating a more complete and representative genome is two-fold: first, GWAS studies would have to identify a diversity of new DNA variants that include historically marginalized populations, we focus on racialized communities in this Comment, but a range of disabled and gender diverse communities must also be considered. Second, they must identify and validate genomic markers that existing catalogs do not already document. If such data were incorporated into the deployment of precision medicine, such a tool might afford a diversity of racialized communities vital access to preventative and diagnostic care for whom the current reality is to typically treat health conditions in acute care contexts [6–10].

Incorporating the genomes of racialized peoples in GWAS studies is not the only issue that confronts the creation of truly inclusive precision medicine. We believe that the following additional question also needs to be asked: how do initiatives which strive to be more inclusive navigate the issue of equity? Scholars examining concerns with equity in precision medicine for racialized communities underscore a range of concerns. These include the balance between individual and group rights over genetic data. Because experiences with the unauthorized secondary use of collected biological samples in past genetic research has discouraged racialized communities from participating in genetic studies, the ownership of genomic data and its governance are also crucial issues relevant to including racialized communities equitably in precision health [11–14]. 

In this commentary, we review the context in which issues of inclusion arose with early genome and genetics studies. We then discuss the productive efforts made to include racialized peoples in more recent human genomics research. In conclusion, we raise critical issues to consider regarding equity in the enterprise of undertaking genuinely inclusive genomics research and precision medicine protocols and commitments as it pertains to racialized communities: we briefly outline such an orientation towards inclusion and equity under the rubric of what we call *Precision Health Equity* (PHE)*.* Specifically, we examine issues of genetic data governance and the terms of participation in inclusive human genomics/precision health research.

### Early human genome projects and “Salvage Genomics”

Launched in the 1990s, the Human Genome Project sought to map out the entire human genome in hopes of identifying disease-causing genetic variants. Though a draft map of the human genome was completed in 2000, new findings and analytical tools suggested that the HGP’s original goal was misidentified because the presentation of disease often involves complex genetic interactions and epigenetic factors (which could be a result of environmental factors). In many cases, disease occurrence for complex diseases, like coronary artery disease or diabetes for example, cannot be reduced to the presence or absence of a single gene variant. (It should be noted however that the opposite can be true for rare diseases, many of which are dependent on single variants.) Despite this, the HGP led to significant advances in DNA sequencing technology and bioinformatics analysis software, that catalyzed an explosion in new methodologies and diagnostics that enabled clinical screening at an unprecedented level. This extended not only to the canonical DNA-based screens (*i.e.* genomic or exomic) but also to other levels of molecular biological assays (e.g. transcriptomic, proteomic, lipidomic, and metabolomic, among others).

Nevertheless, the HGP was limited in the racial and ethnic communities that were included in its original design. Only eleven genomes of predominantly European ancestry were included in the original HGP in response to which the Human Genome Diversity Project (HGDP) was also launched in the 1990s [15]. At the time, the lead team of physical anthropologists and geneticists indicated that the HGDP sought to address a deficit in the diversity of genomes that were not included in the HGP. The study sought to target 722 communities globally whom these scientists believed possessed relatively ‘undiluted gene pools’ due to cultural and geographical factors. Problematically, racialized communities in Africa, Canada, the United States, the Amazon, the South Pacific, India, central America, Siberia, Spain, and Taiwan were targeted for inclusion in the study because they were purportedly from “relatively unmixed descendants of ancestral populations” [16]. The researchers embarked on a program to record humanity’s genetic heritage which might contain the “prehistoric migrations, natural selection, the social structure of populations and the frequency and types of mutations” of the human species based on the faulty assumption that these communities were largely ‘untouched’ by European or other ancestries [16]. Fitting into what social scientists and racialized community members call “salvage genetics”, involving the recuperation of normative and ideological understandings of racial groups who are deterministically associated with genetics and ancestry [17]. The HGDP was criticized for its racist assumptions, for excluding members of the targeted communities from its proceedings, and because of the project’s intention to store the samples and genetic data in repositories and gene banks controlled exclusively by scientists at their home institutions [18, 19].

Sadly, issues of exploitation of different racialized communities, in various global sites, has been foundational in the history of genetics also [20]. In the 1980s, a university-based investigator at the University of British Columbia engaged in unauthorized secondary research on ancestry and retrovirus using blood samples provided by the Nooka (Nuuth-chah-nulth) community in northern British Columbia. The community originally provided samples for a study that would determine if the community possessed a genetic marker for rheumatoid arthritis [21]. The resulting scandal led to the establishment of federal guidelines for medical research involving Indigenous communities in Canada, specifically, the *Tri-Council Policy Statement: Ethical Conduct for Research Involving Humans*, *Guidelines for Health Research Involving Aboriginal People* and *the Canadian Institutes of Health Research Guidelines for Health Research Involving Aboriginal People.* Despite the creation of an ethical framework for biomedical research involving Indigenous communities, the event continues to impact the decisions of Indigenous communities about whether to participate in contemporary genetic screening studies, particularly in western Canada [22, 23]. Knowledge of events like these, and others, circulate within racialized communities also which augment their distrust of human genomics research [24–26]. (While differences between Indigenous and racialized communities must be acknowledged, we also see an opportunity to draw on the insights of scholars working on decolonization and racialized settlers on Turtle Island/North America who suggest that effective racial and social justice enterprises need to recognize the parallel, though not identical, encounters withcolonization and health disparities which often follow as lived realities for both racialized and Indigenous peoples [14, 27]).

Stigmatization of particular groups is also a significant concern because individual genomic data can be correlated with ethno-racial groups if such data were collected over time and analyzed. Once again, it is important to consider previous experiences with genetic screening and group stigmatization. For example, in 1965, the State of California made it mandatory for all Black children to be screened for the sickle-cell *trait* as a way to prevent the disease. Within approximately fifteen years, seven states followed California’s lead requiring newborns be screened and by the late 1980s, prenatal screening for pregnant mothers could also include the sickle-cell trait. But as Troy Duster [24], notes on screening in this period,

“[if approximately ten percent have sickle-cell disease,] more than ninety percent were only *carriers* of the gene, and would live as healthy a life as anyone else….[Therefore,] to screen for a disorder that cannot be cured can tap a reservoir of latent, recurring fears about the motives of those who would [seek to] *prevent* genetic disorder rather than treat it” [24].

While we undoubtedly recognize the significant benefits in using genetic screening tools for preventative and diagnostic care for racialized peoples, we remain concerned that the manner in which screening has been historically conducted with these communities also instructs precision health researchers on its potential pitfalls. The result of an early preventative screening program in California not only stigmatized and notionally tethered the disease to Black Americans when it also occurs among Arabs, Sicilians and other groups with Mediterranean ancestry as Troy Duster’s study elucidates [24]. When screening programs were introduced throughout the United States, a lack of confidentiality of medical records meant that insurance companies could also learn if a child was a carrier of the gene and charge higher rates. Duster’s history also recounts that in the early years, screening was conducted on Black American teenagers before puberty, or it became a requirement before a marriage license was issued or in the event of hospital admission and treatment [24]. Inclusion of sickle-cell in prenatal screening also introduced new pressures on expectant mothers, who typically strongly consider terminating pregnancies when a ‘disorder’ is discovered, thus bringing in concerns about eugenic influences of these forms of genetic screening [24].

### Inclusivity and precision health equity

While the exploitation and harms caused by genetic research are part of a long history of abuses by medical institutions which have deep colonial roots, it must also be acknowledged that some genomicists today are indeed trying to avoid perpetuating inequality and disenfranchisement in human genomics research. These scientists have done so by including racialized peoples in studies in which these communities have, so far, largely been underrepresented [2]. Human genomics and precision medicine require vast and diverse genomic data to effectively analyze complex biological and environmental factors in order to precisely tailor clinical decision-making if a risk-associated variant is found in a patient. A handful of projects have and continue to seek to include individuals with diverse ancestries. For example, the *1000 Genomes* study (2010) sought to create a more complete catalog of genetic markers that were associated with phenotypic variation. It sought to build on previous genome-wide association studies that had gaps when specific regions of the human genome were examined as the locus of a disease-associated genetic variation [28]. We recognize these important efforts because unlike the HGP, *1000 Genomes* includes genetic data from racialized communities while also undertaking measures to recognize the ethical, legal and social implications (which the HDGP did not). Future research in human genomics and precision medicine would benefit from similar measures to include racialized communities however it appears to currently be done in an uneven manner, if at all [3, 4, 15].

When inclusion efforts are undertaken, there are additional precautions to consider regarding how ethno-racial differences are defined and classified. As postcolonial historians of race and caste amply demonstrate, many of the ethno-racial categories that are employed in scientific and social scientific knowledge production, whether it comprises academic scholarship, institutional research or public statistics, originate from taxonomies of ‘native peoples’ globally which were deployed to map out, divide and control peoples falling under European colonization (settler or otherwise) and/or trans-Atlantic slavery [29–32]. These ideologically-derived categories of ethnicity and race have also been coupled with inherited genetic traits, biological conditions and notions of community and nation by both Eurocentric and nationalist-oriented geneticists and scientists in the global north and postcolonial world in the twentieth century [33–35].

Does the adoption and inclusion of peoples who are classified into ethno-racial categories in contemporary precision medicine research reproduce and reify the reductive and segregating effects of colonial-era categories of ethnicity and race? The approach of the UKbiobank and scholars considering this question is instructive. This study strives to construct “a prospective” epidemiological biobank aiming to identify risks to “common, complex diseases such as diabetes, heart diseases, and Alzheimer’s and, it is hoped, providing the basis for improving the diagnosis and treatment of these conditions.” [36]. Influenced by the National Institutes of Health *All of Us* initiative in the United States, the UKbiobank sought to interrogate and adopt ethno-racial categories from the British census, in its sample design while also requiring affiliated researchers to provide data about how the ethnic-racial groups “might differentially benefit from a new experimental treatment or from being affected by a certain disease” [36].

While not solving the problem of reifying these problematic ethno-racial categories, the incorporation of health outcomes in efforts to include racialized peoples in creation of the UKbiobank seems to provisionally demonstrate how communities that are deemed to be racially ‘different’ can be identified for inclusion in human genomics research and precision medicine. Until better conceptualizations of race are generated, the UKbiobank’s approach might also suggest a provisional way to begin to define racialized communities in precision medicine research. We make these two suggestions because the designers and researchers of the UKbiobank appear to reflexively recognize the contested and problematic nature of existing categories of race and ethnicity while still including these communities, and most important, how the biobank’s data may generate data about benefit-sharing and health outcomes that might be pursued thereof with these very communities.

Researchers and bioethicists in human genomics underscore a different, though related, problem of equity which can also impede genuine efforts to fully include and serve racialized communities. Sara Ahmed (2020) interrogates the category of inclusion and (diversity) noting that institutions may adopt discourses of diversity but such alignments “of diversity with institutionality…[may be] maintained only *at the level of appearance”* [37]. Concerning the inclusion of peoples classified as ‘different’ in biomedicine specifically, Steven Epstein (2009) suggests that it is a consequence, in part, of “the biomedicalization” of governing in the United States, namely that inclusion requirements have been incorporated into the Department of Health and Human Services, which funds biomedical research in significant ways, in the context of political advocacy to include members of groups deemed to possess sexual, gender, racial or ethnic ‘differences’ [38]. Here, we direct the reader’s attention to establishing *equitable terms* of including racialized peoples in human genomics research by leading our discussion specifically to open-data sharing practices.

According to geneticist and bioethicist, Krystal Tsosie, open access policies.

“are now widely used by industry agents who have used biomarkers derived from Indigenous communities for corporate profit, while those same Indigenous communities fail to benefit from medical innovations that might improve health outcomes” [39].

To be sure, we examine one constraint to full inclusion and equity though there are likely others also. The fact that large pharmaceuticals fund the collection, and ultimately the generation, of genetic data means that benefit-sharing can be uneven (or entirely absent in some cases) with participant communities. (In fact, this is also part of a concern in biomedicine more generally because similar conditions exist for the production of vaccines, antibiotic treatments, antivirals among other treatments and interventions.) While we recognize the role of bioscience organizations and pharmaceutical actors as important stakeholders working in human genomics and precision medicine, we also support the continued exploration of different or alternative arrangements and agreements with participant communities than what predominates at present.

Existing models might point to ways forward while also addressing the attendant issue that many large-scale human genomics studies are publicly-funded and therefore provide benefits for broad public health while also addressing inclusion and equity goals for racialized peoples (who have been, it is important to note, historically excluded in favor of perceptions of broader public health priorities) [12, 20, 24]. It should be noted that many of the models which have already been proposed address research in human genomics involving Indigenous communities in Turtle Island/North America, Aotearoa/New Zealand and Australia (among other regions).

Keolu Fox [40] writes in the context of the participation of Indigenous communities in human genomics studies,

“One way to facilitate a paradigm shift toward equitable benefit sharing would be to ensure that Indigenous people have control of data from Indigenous populations, including digital sequence information. Two approaches for achieving this control have been used: individual-interest models (also known as shareholder models, which involve fractional ownership of stock) and collective-interest models (which involve community trusts). LunaDNA, a community-owned platform for biomedical research, is an example of the fractional-ownership model. This public-benefit corporation distributes proceeds from the platform back to people who share their DNA for research. Community trusts, which not only provide subsidized access to drugs but also reinvest in communities that participate in genomics research, can also be established in partnership with both the NIH and pharmaceutical companies” [40].

This intervention raises two specific questions which are central to data governance in human genomics and precision medicine research that involves racialized peoples: a) does a project adequately partner with and execute a data-sharing agreement, along with the requisite governance structures that such partnerships and agreements require, with its racialized participants? and b) are measures undertaken to ensure that racialized communities are, or will be, beneficiaries of the therapeutics derived from these collected genomic data?

Scholars, as well as institutional initiatives, working towards racial and social justice have proposed additional productive strategies to consider when striving to address the problem with which Fox engages, namely data governance and self-determination of historically colonized and racialized peoples; such strategies might be a productive starting point in our view. For example, The First Nations’ Information Governance Centre (FNIGC) in Canada, that has articulated the OCAP® (Ownership, Control, Access, and Possession) framework for data governance offers us a model for equitable principles for Indigenous data governance which could be adapted to human genomics research involving racialized communities [32].

As such, an approach embracing PHE might pursue the incorporation of racialized participants on terms that are equitable which means going beyond the typical requirements of university-based research ethics committees. The application of a template of equitable data governance does not inhere PHE; instead, we suggest the co-creation of governance protocols, structures, and timelines through a partnership between racialized community members and researchers, perhaps also including policy makers and other entities funding such research–a set of approaches to biomedical research already endorsed by scholars working on genetics and health equity for racialized communities. In the early 2000s, alternatives to conventional biomedical research have been delineated in the context of genetic screening studies conducted specifically with Indigenous communities, namely an approach advanced in Canada by Laura Arbour and Doris Cook referred to as *DNA on Loan* [23]*.* We believe that there is merit in, and PHE is characterized by, the adaptation and extension of insights from these studies to contexts in which racialized communities *choose* to engage with precision medicine with respect to the following considerations. Whereas typical research design involving local communities may formulate its questions largely in reference to extant academic insights in a specific field, approaches aligned with PHE and the community-based participatory research principles on which *DNA on Loan* is based, emphasize forming a research partnership, indeed investing in longer-term relationships, with communities included in research studies [41]. Within such a context of establishing a more equitable relationship, both researchers and community leaders and health advocates can formulate research questions and ultimately design a project that is aligned to their health priorities in addition to their cultural understandings of disease, cure and wellness. Because genetic screening is typically prospective, collaboration between researchers and communities allows the latter to consider the risks they are willing to undertake, i.e. if disease-associated markers are discovered and how they might contend with reproductive issues and possible stigma [23]. To be sure, research projects are also designed to address the development needs of participating community, offering its members opportunities for training (that, in turn, produces new productive relationships between researchers and community members in relations to knowledge production, and therefore, power) [39, 42].

As a consequence, collaborative genetic research which comprises PHE requires negotiation and agreement on a governance structure with the participating community and discussions about the mode and frequency of reporting research progress and results back to the community [43, 44]. With the possibility of storing genetic samples for future study or analysis, referred to as *biobanking,* specific agreements on genetic samples and data governance are indispensable to equitable inclusion of racialized peoples in the genomic science that undergirds precision medicine. Genetic samples and the possible data derived from them can be of cultural significance to racialized communities. Similar to accounts about the sacred nature of blood and DNA to Indigenous communities in Canada or the United States, many racialized communities from the global South—differentiated as they certainly are—attribute special (and varying) meanings to blood and tissue also [22, 45, 46]. Nevertheless, the benefit of access to preventative and diagnostic care which precision medicine can possibly offer makes it of interest to some, but certainly not all, racialized communities based on their health priorities, cultural understandings of health and wellness, and collective decisions [8, 22, 45].

In the North American context, approaches in genomic-based studies derived from *Indigenous Data Sovereignty* (*IDS* below) suggest some decolonial paths forward though, again, no single approach or template can be assumed given the diversity of historical contexts, interests, and institutions among participating racialized communities. *Silent Genomes* (SG) in Canada, the Native Biodata Consortium located on the sovereign lands of Cheyenne River Sioux Tribe in Eagle Butte (South Dakota), and *Aotearoa Variome* (AV) in Aotearoa/New Zealand manifest IDS principles especially as it concerns data governance. [8] The genetic screening components in each of these initiatives are grounded in the cultural ethics of the participating community thus characterizing the ethos with which data governance is designed. In the AV project for example, DNA is interpreted as takoha, or as a gift of responsibilities, that obliges data users to use it in a culturally-appropriate manner and in the service of the community’s needs [22].

The decolonial commitments embodied in these initiatives are reflected in their institutional structures as well. They are co-designed and co-led by Indigenous scientists and community members as a way to commit to “Good Actions/Actors” [22]. For example, at the time of writing, many Indigenous partners collaborate in the SG studies, since eight communities are involved, and the creation of an Indigenous genomic biobank continues to be explored. These nations continue to consider issues such as the implementation of a genomic biobank and ensuring it can be sustained as a clinical tool which can improve health outcomes within the participating communities. To this effect, an International Indigenous Genomics Advisory Council oversees the development of a governance structure for the database and best practice policy models for the oversight of biological samples and genomic data [22]. One hope is that such a body may also facilitate the identification of the clinical focus of a genetic study (genetic conditions occurring among children are the focus for SG for example). Samples are stored in a biobank or repatriated to the participating community for disposal in a culturally appropriate manner. OCAP® principles (Ownership, Control, Access, and Possession) developed by the First Nations Information Governance Centre, define the parameters for First Nations data governance, but may also be useful for other Indigenous and as stated above. In the case of the Native Biodata Consortium, Indigenous scientists work with communities and act as stewards of research samples and data keeping them “within the provenance and governance of Indigenous communities” participating in the studies [39].

To fully explore the approaches described in this Comment, it is important to underscore the centrality of training researchers and clinicians in practices that promote equity in the context of human genomics and precision medicine. Currently, some medical schools have adopted learning modules which examine health disparities, social determinants of health and stress the importance of equity and inclusion for equity-seeking groups like racialized peoples [9, 47]. We seek to complement these efforts by highlighting the issues of health equity as human genomics research expands and informs multiple fields in the life sciences. We suggest a mode of education that couples PHE-informed human genomics research with training. Mentioned above already, this might begin with capacity building within racialized communities by partnering with them and extending to its participating members opportunities for training in human genomics. Of course, this also relies on initiatives to create a robust pipeline for training members of racialized groups in the life sciences more generally (i.e. inclusive medical education, a topic that is relevant to PHE but beyond the scope of this particular essay). The H3Africa Consortium exemplifies the kinds of commitments which comprise PHE in the context of training and capacity building. The H3Africa Consortium was the first major pan-African study to study a wide variety of communicable and non-communicable diseases and traits across the continent [3]. The Consortium is committed to “the dissemination of bioinformatics skills, and design of genotyping array and analysis tools.” as well as the development of bioethics, community engagement, data sharing and governance and disease awareness in human genomics research [3]. Adopting this approach as a point of departure, we envision equitable human genomics research, that is aligned with the orientation of PHE, comprising the coupling of the research and clinical enterprise to training in postcolonial science and medical history, in addition to health disparities and health equity research, among participating community members.

## Conclusions

While no single IDS initiative ought to be used as a template, those already in existence advance models of how precision medicine might be adopted in manners that are both inclusive *and* equitable, while also being adapted to a diversity of contexts, values and needs of different racialized communities. Stated in the broadest sense, full partnership in a given genetic study and ownership over the biological samples and genetic data derived from genomic researchs characterize central tenets to Precision Health Equity for racialized communities which merit further and deeper exploration in specific human genomics and precision medicine initiatives that seek to include racialized communities.

## Data Availability

Not applicable.

## Abbreviations

AV: Aotearoa Variome
FNIGC: First Nations Information Governance Centre
GWAS: Genome-wide Association Studies
HGDP: Human Genome Diversity Project
HGP: Human Genome Project
IDS: Indigenous Data Sovereignty
OCAP®: Ownership, Control, Access, and Possession
SV: Silent Genomes
